# The global movement towards a public health approach to substance use disorders

**DOI:** 10.1080/07853890.2022.2079150

**Published:** 2022-07-06

**Authors:** Kimberly Johnson, Irina Pinchuk, Marie Isabel E. Melgar, Martin Osayande Agwogie, Fernando Salazar Silva

**Affiliations:** a International Consortium of Universities for Drug Demand Reduction, University of South Florida, Tampa, FL, USA; b Institute of Psychiatry of Taras Shevchenko National University of Kyiv, Kyiv, Ukraine; c Ateneo de Manila University, Quezon City, Philippines; d Global Initiative on Substance Abuse, Lagos, Nigeria; e Universidad Peruana Cayetano Heredia, San Martín de Porres, Peru

**Keywords:** Global, substance use, addiction, opioids, policy, public health, law enforcement

## Abstract

Drug misuse is a global problem. Markets that supply illegal drugs often span international borders. However, each country has different primary drugs of use, populations that are using and consequences of use. The policy approach of each country to addressing substance use disorders can be characterized along a continuum between purely public health approaches and purely law enforcement approaches. Historically, a law enforcement approach has been the primary strategy in much of the world. However, there is a growing movement towards use of a public health approach. This article provides four case examples, Ukraine, Philippines, Nigeria and Peru, where there is movement to develop addiction public health infrastructure. The work varies by country, but includes regulatory changes, workforce development and resource allocation all of which are supported by the United Nations Office on Drugs and Crime (UNODC) and multi-national organizations that provide training and technical assistance, funded primarily by the European Union and United States governments. All four countries highlighted have barriers to moving towards a more public health approach which may include popularity of the law enforcement approach, turbulent government environments, and economics of being a drug producing nation. However, whether starting from the top down with changed policies, such as Ukraine or from the bottom up with training community members as in the Philippines, each country provides an example of how donor resources can be applied to make the transition towards a more humane and evidence-based approach to addressing substance use disorders.

**Key Messages**
1. While the primary approach to addressing drug use has focussed resources on law enforcement for over 100 years, many countries are adopting elements of a public health approach including prevention and treatment of the harms of drug use including substance use disorders.
2. There is a growing global movement to make policy towards drugs and drug users more humane and evidence-based.
3. Donor nation resources can be applied in a variety of combinations to improve care and outcomes for people who use drugs in low- and middle-income countries.

## Introduction

While drug misuse is a global problem and the markets that supply illegal drugs are international in nature, each country has its own specific issues in terms of drugs of use, populations that are using and consequences of use. The United Nations Office on Drugs and Crime (UNODC) 2021 report highlights the differences such as opioid overdose deaths in the USA, injection drug related HIV transmission in Eastern Europe, methamphetamine use in Southeast Asia, the use of pharmaceutical opioids and increased transnational trafficking crime in West Africa, and cocaine use and trafficking in South and Central America [1]. These differences in drug related problems lead to differences in policy focus and interventions that are promoted in each country. While drug markets are international, policy efforts including the choices of the distribution of resources among possible interventions is local.

## Law enforcement versus public health approach

The policy approach of each country to address substance use disorders can be characterized along a continuum between purely public health approaches and purely law enforcement approaches [2].

Western Europe is seen as the region with the strongest emphasis on a public health approach to addiction [2]. The European Monitoring Centre for Drugs and Drug Addiction (EMCDDA) formally established in 1995, was created to support the European Union region in a unified response to the drug problem. It focuses on providing epidemiologic data for the region and supporting the use of evidence-based policies and practices across the public health and public safety continuum. Western Europe is perceived as the place where harm reduction strategies, initially introduced in response to the HIV epidemic here as in other regions, had the broadest adoption [3].

The law enforcement approach in much of the world is generally attributed to the United States and the “War on Drugs” initiated in 1975 [4–6], though the origins of an international focus on law enforcement as a strategy go back as far as the original international treaties in 1912 [5]. The heavy investment by the United States in supply reduction in Southeast Asia and South and Central America led to these regions skewing towards the law enforcement end of the continuum. Other countries, like Russia and Eastern European countries that were once part of the Soviet Union have also had a historical focus on a law enforcement approach [7]. In countries with a primarily enforcement approach, drug users are often treated as criminals who need to be punished rather than patients who need to be treated.

While some might characterize the public health approach as European and a law enforcement approach as American this is an oversimplification as the E.U. and the U.S. both use and export elements of both [4].

The HIV epidemic in Europe, sub-Saharan Africa and Southeast Asia led to the introduction of medication for opioid use disorder, particularly methadone, as a strategy to reduce transmission of HIV from IV drug users. Resources to cover the cost were provided by international donor organizations including the E.U. and U.S. governments [8–10].

In Latin America, the movement towards a public health approach has been precipitated more by the recognition of the failure of the law enforcement approach and its impact on the stability of government in the region [11]. Narcoterrorism, a term first used in Peru regarding drug traffickers use of violence against drug enforcement officers, and later referencing the various links between drug trafficking and terrorism and civil unrest [12] and corruption [13] have been associated with the law enforcement approach towards drug use. While public support for a law enforcement approach remains high in some countries, some governments and those in academia and healthcare are recognizing the need for a transition to a more humane, evidence-based public health approach [14].

## Human rights perspective

Significant human rights abuses against drug users are not limited to low-income countries or countries with autocratic forms of government. The militarization of policy activity regarding drug use, the use of extreme force and violation of rights to privacy in particular, have been well documented in the United States [for example 15,16].

The historical context of a primarily law enforcement approach to addressing drug use and people who use drugs in most of the world, the militarization of policing efforts against drugs in many countries, and abuse of patients in treatment for drug use may lead to a pessimistic view of the international effort to reduce the harms of drug use [4]. Against this backdrop, there is an international effort to reduce human rights abuses and take an evidence-based approach to addressing substance use and its problems. This can be seen by the recent UNODC Commission on Narcotic Drugs Ministerial Declaration where the United Nations affirms the goal to protect human rights and to use a balanced, evidence-based approach to the world drug problem with a focus on using data to drive policy recommendations [17].

The UNODC strategic plan for 2021–2025 makes a commitment to improve coverage of effective prevention and treatment through promotion of evidence-based services *via* two standards documents that have been created in conjunction with the World Health Organization (WHO) and with the input of multinational groups and individual experts [18]. The treatment standards are designed around broad principles of availability, efficacy, ethical practice and responsiveness to patient need. The prevention standards are organized around participant age and intervention target population with a rating for the level of evidence for each intervention described [19].

## Efforts to support international standards adherence

Significant training and technical assistance to governments and others are offered through UNODC, the E.U. and United States governments and international non-governmental organizations (NGOs). Included in these efforts is TreatNet, a training programme on evidence-based practice (EBP) in addiction treatment, and the Universal Treatment Curriculum (UTC) and the Universal Prevention Curriculum (UPC) offered by the Colombo Plan along with the Global Centre for Credentialing and Certification (GCCC) that provides individual provider certification processes that governments can adopt, and the United States Agency for International Aid (USAID). Training and technical assistance that does not necessarily run through government agencies is provided to university faculty through the International Consortium of Universities for Drug Demand Reduction (ICUDDR), to individuals through the International Society of Substance Use Professions (ISSUP), to communities through Community Anti-Drug Coalitions of America, and to governments, and organizations through the International Technology Transfer Centres, all funded at least in part by the U.S. State Department.

While incorporating this broad international perspective, each U.N. member nation continues to adopt its own set of policies and design its own approach that addresses the local context. For that reason, it is useful to examine case examples where the approach to drug use is shifting towards a more evidence-based policy and programme focus. Following are examples of four countries: one from eastern Europe (Ukraine), one from Southeast Asia (Philippines), one from sub-Saharan Africa (Nigeria) and one from Latin America (Peru). These countries have been chosen as a convenience sample to highlight the differences in approach to implementing humane, evidence-based policy and practice. While Ukraine has a top-down policy approach that is precipitated by its desired entry into the European Union [20], the Philippines has a bottom-up approach focussed on work force development in the shadow of a punitive public policy. Nigeria, as an emerging economy, a transit nation and more recently a producer nation is experiencing new drug use problems that need solutions [21]. Peru, as producer nation, has a long history of drug cultivation and use. Revenue from the drug trade and from the United States government’s efforts to eliminate it are contributing factors to government instability and history of domestic terrorism [22]. Barriers to a more evidence-based approach are similar to those of Nigeria and Philippines, but more embedded in history and culture. Our goal in selecting these case studies is to portray the variety of issues and approaches that are being used to reach a common outcome of a more effective national strategy to address drug use. While there is a global effort to improve policy and practice towards addressing drug use, each country has unique features that require a country-by-country approach.

## Country case examples

### Ukraine

Ukraine is the second largest country in the European region. It has a population of 44 million [23]. After achieving independence from the Soviet Union in 1991, Ukraine experienced political upheaval for a number of years, including the “Orange Revolution” in 2004 and the “Revolution of Dignity” in 2014 [24]. The invasion by Russia of Crimea and eastern Ukraine, begun in 2014, led to a humanitarian crisis with about 3.4 million people in need of humanitarian assistance, and about 1.4 million people internally displaced. In the occupied territories, there was a reversion to punitive treatment of drug users and closure of opioid substitution treatment (the term used in the country that is a synonym for medication for opioid use disorder in the United States) in the regions [25].

Within this context, Ukraine had an increase in the use of stimulants and new psychoactive substances [26]. There is a high prevalence of substance use disorders) and low treatment coverage, especially for alcohol use disorder [27]. Opioid use and injection drug use is a primary driver of HIV incidence in the country with 25% of new infections linked to people who inject drugs most of whom inject opioids [28]. Illicit methadone, produced in clandestine laboratories, has been the principal opioid used by injection drug users in Ukraine [26]. The gender ratio of substance use is gradually levelling off among adolescents, but the prevalence of substance use disorders remains significantly higher among men [29].

Ukraine's desire for integration into the European Union, and agreement to participate in the implementation of the United Nations Sustainable Development Goals required the improvement of policies to address drug use in accordance with the E.U. model. From 2013 to 2020, Ukraine had a “Strategy for State Drug Policy”, which did not achieve its goals because, like many countries they were overly ambitious and poorly funded, but also because of conflict between government agencies regarding approach [20,30]. Government reorganization impeded implementation of some activities and there was insufficient coordination within and between ministries. For example, the drug monitoring function was reorganized three times between 2013 and 2020. This has led to a loss of human resources, followed by a lack of consistency in the collection and preparation of the Report on the Drug Situation in Ukraine which is provided to EMCDDA.

Built on the Soviet model, the Ukraine addiction treatment system has relied heavily on addiction psychiatrists. The number of addiction psychiatrists has decreased every year, and the percentage of non-medical personnel in public institutions remained consistently low [31] creating structural issues in access to care. In private institutions, there are more non-medical personnel. Their need for training is high; however, the number of training programmes at public universities is still small and is mainly focussed on physicians [31].

Prior to the most recent invasion by Russia, a new ‘Strategy of State Drug Policy until 2030’ had been developed, and was awaiting approval by the Government of Ukraine [32]. This strategy was based on the provisions of the constitution of Ukraine, national legislation and relevant international legal instruments of the UN, Council of Europe and the E.U., including the Convention for the Protection of Human Rights and Fundamental Freedoms, the Convention for the Protection of Human Rights and Dignity in Biology and Medicine. Under the proposed policy strategy, treatment entry cannot occur without patient consent. The strategy stated that mental and behavioural disorders due to the use of psychoactive substances create significant risks to public health and welfare as well as being detrimental to the economy and national security. This proposed strategy indicated an effort by the government of Ukraine to recognize the need for evidence-based interventions and to address human rights abuses towards people who use drugs [33].

There are ongoing efforts by multinational NGOs to increase access to treatment, particularly opioid substitution therapy in Ukraine. Opioid substitution therapy only became available in 2004 with the introduction of buprenorphine and 2007 when methadone was first used medically [34]. UNODC, United States Agency for International Development (USAID), and the United States State Department Bureau of International Narcotics and Law Enforcement Affairs have all invested heavily in improving access to care in Ukraine through both direct support and training and education to policy makers and treatment providers. In preparation for implementing the new strategy, a mapping of training needs for addiction treatment professionals and paraprofessionals was completed [31]. Additional activities in support of quality improvement and work force development include participation in the ICUDDR and the creation of an International Technology Transfer Centre based on the U.S. Addiction Technology Transfer Centre model (Table 1) [35]. These activities engage universities in provision of pre-service and post-service training. They extend early investment by Colombo Plan and UNODC in training and provide a sustainable model for transferring knowledge from within-country experts [31].

**Table 1.** Work Force Development supports by international organizations.

Country	Regulatory change	In country resource allocation	Workforce development	TreatNet	Colombo plan	ICUDDR	ISSUP	CADCA	ITTC	USAID
Ukraine	x	x	x	x	x	x	x		x	
Philippines			x		x	x	x	x		x
Nigeria	x	x	x	x	x	x	x		x	
Peru		x	x	x	x	x	x	x	x	

The invasion of Ukraine by Russia in February 2022 has created disruption in all of the planned service system changes. Most of the methadone manufactured in Ukraine was made in two factories, one in Odessa and the other in Karkhiv but both have stopped production [36]. The pandemic changed the distribution of methadone from a clinic based daily activity to a prescription-based system for up to 30** **d of medication. This system change was of use when the invasion started. Physicians wrote prescriptions for patients before they evacuated from the country during the invasion. Many patients have prescriptions, but are unable to fill them because even in the areas of the country that are not affected by Russian bombing, the medication is not available. Multiple international organizations are trying to ensure methadone and buprenorphine availability in the country along with other vital medications and food [36]. Addressing health needs for refugees with chronic conditions, including substance use disorders, is a priority for most of the bordering countries where Ukrainian citizens have sought refuge [36]. Some addiction psychiatrists have remained in the country to provide medical care for the wounded. While focus is currently on the immediate needs of fighting a war, the Ukrainian addiction work force and the officials that led the change effort express a desire for a return to systems building efforts that were underway before the pandemic and the invasion of the whole country. Plans to continue training of professionals and paraprofessionals *via* distance learning are in process [37].

### Philippines

The Philippines Dangerous Drugs Board (DDB, a government agency) survey reported an estimated 1.67 million people who use drugs in 2019 [38]. The survey reported that lifetime drug use prevalence among Filipinos age 10–69 was 5.8%. Methamphetamine, known locally as Shabu (47. 9%) and cannabis (35%) were named as the most common substances used by survey participants [38]. The total number of reported people who use drugs dropped more than 50% from 2016, the first year of President Duterte’s war on drug users.

The current law enforcement approach of the Philippines government towards reducing drug use has received much international attention. The anti-drug campaign was articulated through ‘Project Double Barrel’, a two-pronged framework with a different approach for people who sell versus people who use drugs. The two approaches are termed Operation High-Value Target (HVT) targeting ‘big time drug sellers’, and Operation Tokhang, which targets ‘small time drug sellers’ and users [39].

Operation Tokhang, loosely translated as ‘knock and plead’ programme, has law enforcement going to the homes of people who are suspected of using drugs or of low-level drug offences and offer them the choice to surrender and be sent to rehabilitation facilities rather than prosecuted or murdered for drug crimes. Over a million people have surrendered. Surrenderers that complete a community based out-patient treatment programme are eligible to have their name removed from the watch list.

Efforts to ensure that surrenderers and others who need treatment are able to access it have been supported by international organizations. Provision of technical assistance to duty bearers through the assistance of USAID, UNODC, the Colombo Plan Drug Advisory Program and ISSUP, have been instrumental in training local people on evidence-based treatment and prevention interventions. USAID is currently funding a national initiative to start and expand the community-based drug rehabilitation programmes in several cities in the Philippines. The vision of this project is that by the end of 5 years, more communities will be able to provide evidence-based community-based drug rehabilitation services that people who use drugs and their families can access without fear, and that the country will have infrastructure in place to sustain ongoing workforce development activities to improve the quality of care and respect for the human rights of people who are in treatment programmes.

The content of community-based drug rehabilitation programmes was adapted from training materials created by UNODC combined with materials developed for the United States. The materials were adapted for cultural context, simplification for non-literate patients and providers, language and the specific systems in place in the Philippines. While implementation and evaluation are ongoing, there is some evidence that these modified programmes have improved outcomes for people who use drugs in the Philippines [40–42].

In 2020, the University of Philippines, Manila created a fellowship in addiction medicine, the first in the country. The College of St. Benilde offers a short-term diploma programme in addiction counselling. International organizations are supporting training of faculty and encouraging the development of programmes and courses on addiction in professional education for medical and other professions. These two schools as well as others that offer courses within public health or psychological training participate in international activities *via* ICUDDR and ISSUP. Sustainability of the USAID funded efforts is supported by engagement of Community Anti-Drug Coalitions of America in developing community coalitions that focus on prevention of drug use and like Ukraine, through involvement in international training opportunities through the various NGOs that offer it.

The DDB includes in its responsibilities a programme on drug education and prevention. They offer preventive programmes for people of all ages. Many of these interventions are implemented nationwide. The prevention efforts at the community level, in recent years, have been intensified through the multi-agency collaboration among the Dangerous Drugs Board, Department of Health, local government units, academia, NGOs, the church and community-based organizations.

Both government and private drug treatment facilities co-exist to accommodate the volume of people who have been mandated to treatment. The DDB facilitated a systematic client flow diagram to illustrate the pathways through the treatment system at the community level including medical and psycho-social interventions. However, the COVID-19 pandemic has had an adverse effect on treatment availability. Adjustments to virtual and tele-counselling have been made, but in areas of the country with low bandwidth, these solutions are inadequate. Outpatient programmes were transformed to telephone session and occasionally followed up by home visits. Family therapies and psycho-education were conducted through Zoom. During the pandemic, the duration of an in-patient recovery programme still ranges from 3 to 6** **months or longer. Clients often choose a shorter stay because of the prohibitive cost of treatment. As in other countries, there have been fewer in-patient admissions during the pandemic [43].

These efforts to expand the use of evidence-based prevention and treatment interventions and to increase both the number and skills of people working in prevention and treatment in the Philippines may seem in stark contrast to the stated government policy. However, they have developed largely in response to the current political strategy and though financially supported by aid organizations, have the support of communities and many in congress, local government and law enforcement. Training and programme development have continued after a brief hiatus at the beginning of the pandemic [40,41].

### Nigeria

According to the first comprehensive national drug use survey conducted in Nigeria, 14.3 million adults aged 15–64 (14.4%) used at least one psychoactive substance (excluding alcohol and tobacco) in the previous year [1]. This figure is considerably higher than the most recent global annual prevalence rate (5.6%) of all substances used among the adult population [1]. In addition, among this 14.3 million people, 20% have a diagnosable substance use disorder, a figure that exceeds the global average by 11% [1]. One in five high-risk (defined as those who used opioids, cocaine or amphetamines five times in the 30** **d previous to the survey, or those who injected drugs) persons who use psychoactive substances injects them; pharmaceutical opioids account for the most injected substance. As a country, Nigeria is about 3% of the world’s population, but account for 6% of the world population of cannabis users and 14% of the world’s population who misuses pharmaceutical opioids, particularly tramadol and cough syrups containing codeine or dextromethorphan [1,44,45].

Efforts to address the drug problems in Nigeria started in 1935 with The Dangerous Drugs Ordinance [46]. This was followed by The Indian Hemp Decree No 19 of 1966. Under this decree, cultivation of cannabis could lead to 21** **years imprisonment or death penalty and smoking cannabis led to a mandatory sentence of 10** **years imprisonment [44]. Since then, amendments to these laws have increased penalties, stipulating death penalty by firing squad for any person selling or using cocaine or other similar drugs without lawful authority. The Nigerian drug policies have, therefore, been described as containing some of the most draconian provisions ever applied to eradicate drug trafficking and use [47].

In 1989, The National Drug Law Enforcement Agency (NDLEA) was established as a unique agency with the dual responsibilities of drug supply suppression including arrest, seizures and prosecution as well as drug demand reduction including prevention, counselling and after care [48].

Nigeria is a producer country of both cannabis and more recently, methamphetamine. Methamphetamine is produced in clandestine drug laboratories leading to increased availability and accessibility of methamphetamine for local consumption in Nigeria and neighbouring African countries [21].

Local studies have shown that criminalization has not reduced substance use, rather it has produced social harms including stigma, large proportions of the population with criminal records, disruption of relationships, loss of employment and housing and drug-related violence [49,50]. It has also prompted people who use drugs to adopt more dangerous practices such as using in riskier settings where social controls are weak and substances are more likely to be adulterated [51].

Despite the challenges of substance use in Nigeria and Sub-Sahara Africa, treatment services are scant. Service providers in various fields that encounter people at risk or who use drugs do not have adequate education about substance use disorders or evidence-based interventions to prevent or treat them [52].

In the past decade efforts to address drug use from the public health perspective have been implemented with the support of UNODC through the E.U.-funded project, ‘Response to Drugs and Related Organized Crime in Nigeria’. Through this project, the major stakeholders in drug control in Nigeria including the NDLEA, the Federal Ministry of Health, and NGOs have received institutional support and capacity building for the workforce. This includes the designation of 11 drug treatment centres as training hubs; the development of national minimum standards for drug dependence treatment in Nigeria; national guidelines for the treatment of substance use disorders for Nigeria; a national policy for controlled medicines and its implementation strategies [49]. After a negative review by international evaluators, the NDLEA counselling facilities increased staffing and implemented a Standard Procedures and Practice Guidelines for counsellors. Drug treatment data collection was instituted with the development and utilization of training manuals such as the TreatNet modules [53], and the Unplugged school-based prevention programme [54,55]. Similarly, the introduction of the UTC for Substance Use Disorders [56] to Nigeria by the Federal Neuro-Psychiatric Hospital Yaba, Lagos state and the Federal Ministry of Health as the focal points and the introduction of the UPC for Substance Use Disorders by Global Initiative on Substance Abuse as the focal point have significantly improved the capacity of the prevention and treatment workforce in Nigeria [51,57]. These two standardized curricula, developed with funding support by INL have been deployed as training tools and in academic programmes globally [58,59]

The increased membership and interest of drug demand reduction practitioners in ISSUP and ICUDDR have further exposed practitioners to international knowledge on evidence-based service delivery. This has also increased universities involvement in the development of academic programmes. For example, before the introduction of ICUDDR to Nigeria in 2018, no academic programme, from certificate/diploma to postgraduate degrees, focussing on drug issues were offered in any university in Nigeria [58,60]. This has since changed with the introduction of post-graduate programmes in addiction studies in two Nigerian Universities, Niger Delta University, in Bayelsa state and Nnamdi Azikiwe University, Awka, in Anambra state. These universities are set to commence post-graduate programmes with many more working to establish similar programmes with implementation support from ICUDDR. In sum, Nigeria, with the support of international donors, has created a more humane treatment infrastructure and begun to develop a work force with skills in providing evidence-based treatment and prevention interventions, while still leaving in place the statutes that severely punish people who use or sell drugs.

The pandemic reduced treatment use in Nigeria, especially by women [50]. Community-based services moved to video and telephonic support services and delivery of medication, but many people were not able to access services due to connectivity issues [50]. Service use rebounded once lockdowns were lifted, though the number of women in treatment remained low compared to before the pandemic [50]. Activities to improve the quality of service including training activities moved online as well, and while connectivity was less of an issue for professionals, issues with connectivity and power availability impacted the ability of professionals to fully participate.

### Peru

According to the Report on Drug Use in the Americas 2019 [61], Peru has a relatively low prevalence of alcohol and drug use compared with other countries in the region, though the data for use in the adult population is over ten years old [61]. The rate of past month alcohol consumption was 31% in 2010, the most recent year of data available, and past year marijuana use at 1% of the population [61]. However, Peru is second only to Chile in its use of cocaine base paste (CBP) which has a past year prevalence of use of 1.5% in the population. CBP is an intermediate product of the production of cocaine, the use of which was first identified in Peru in 1972 [21].

Peru is one of the primary national producers of cocaine and coca products and its policy has evolved within that role in the drug economy [22]. Historically, in Peru there is an association between the Andean man, work, and the coca leaf. The Andean cultures have used coca leaf, attributing magical properties to it, and recognized its ability to mitigate hunger, cold and physical fatigue during workdays in the fields and mines [62]. Currently, it is believed that consumption of chewed coca leaf in rural areas remains highly prevalent among agricultural workers, though data is lacking. The cultural heritage surrounding the use of coca has rendered eradication programmes or alternative development programmes ineffective, though these are the primary historic approaches to addressing use of CBP and cocaine in the country and region [22].

While Peru’s national drug plan includes prevention and treatment, these two activities compromise only 4.3% of the drug policy budget [63]. It was late to participation in the drug wars, but its policy for the past 20** **years has focussed on supply reduction and meeting U.S. demands for coca eradication [22]. Peru has some of the heaviest penalties in the region for trafficking and its eradication programme is primarily a military exercise, though it does not criminalize drug use and in 2017 legalized medical marijuana [63]. As in the Philippines, there is widespread public support for harsh penalties for drug-related crimes [63].

In Peru, availability of training in treatment and prevention interventions based on scientific evidence has increased in the past decade, especially since the launch of the UTC in 2014 and the UPC in 2015 [56,64]. The introduction of these curricula to the country began by sensitizing political decision-makers regarding the fact of an evidence base for treatment and prevention intervention. Much like its origins in the United States, treatment in Peru has been historically provided primarily by people in recovery based on their own experience.

Based on the sensitization training they received, government officials provided approval for instituting training and quality improvement activities within treatment programmes, but did not make regulatory changes. These curricula were also adopted in some universities in the region so that people working in treatment programmes can be trained pre-service. Since 2018, a specialized training programme for the treatment of women, based on the Guiding Recovery of Women, a module of the UTC, has been developed and a diploma programme in School Prevention, based on the UPC, have been implemented at the Universidad Peruana Cayetano Heredia. The first graduates of both programmes have begun working in the field.

Efforts to introduce evidence-based interventions continue to grow in the region and in Peru specifically, and an understanding of scientific evidence as a basis for quality and effective interventions is increasingly accepted and adopted by policy makers as evidenced by participation in policy maker training and approval of training and technical assistance to ministries of health, universities, and individual health care workers [65]. Faculty from Peruvian universities have begun training faculty in Paraguay and Colombia on using these training curricula in education programmes and in designing education programmes in addiction prevention or treatment studies. They are now not only the recipients of training resources, but the deliverer of them to other countries in the region.

In late 2021, Peru created an International Technology Transfer Centre that is actively engaged in needs assessment for the country and developing mechanisms for assessing needs that does not rely on expensive, once per decade, national surveys.

## Discussion

**Figure 1.**
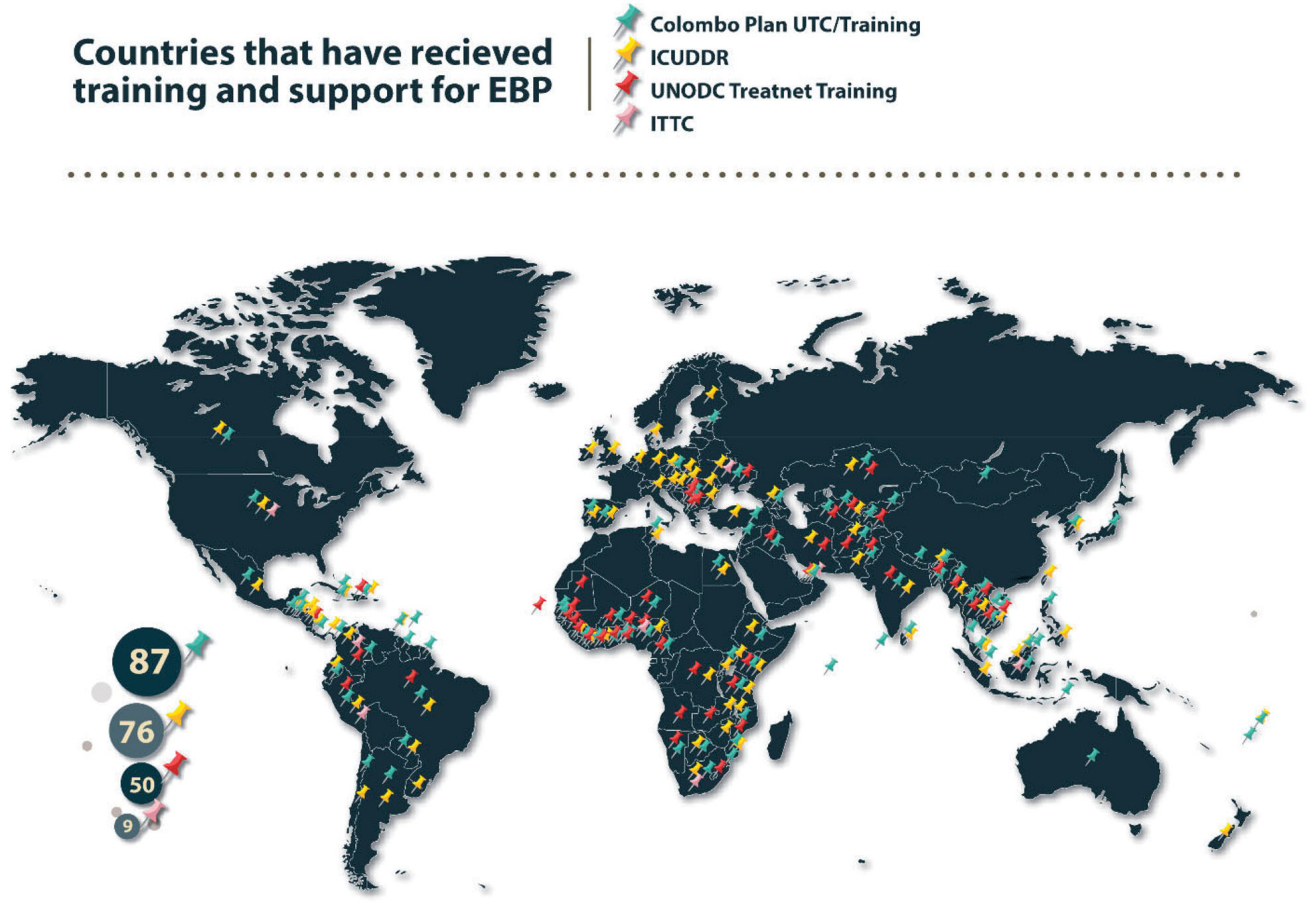
Countries that have received training and support for implementing evidence-based practices to address substance use disorders.

Addressing drug use *via* attempting to reduce the supply through a law enforcement approach has proven costly and less effective than hoped, both within the United States and internationally [66], yet it remains the dominant strategy in most of the world [4]. Efforts led by the United Nations, European Union, and international NGOs have highlighted the negative effects of a singularly focussed law enforcement approach including militarization of law enforcement, human rights abuses, corruption and increased crime [67,68] and fostered a global movement towards an evidence-based public health approach instead. The goal of this study, was to use case examples to highlight the emergent movement to shift to a focus on evidence-based, humane public health approach that includes prevention, treatment and harm reduction strategies. While the map in Figure 1 displays the breadth of the international effort, the case studies provide a concentrated view of the various means by which this effort has engaged with different countries to adapt to specific histories, current situation and expected needs.

These four examples from countries from different regions demonstrate the variety of pathways towards a transition in approach towards drug use. Each country, for different reasons, has a historically law-enforcement approach. Law enforcement remains a strong element in approach for all of the examples provided here. Even Ukraine, where a more European model of making possession of small amounts of drugs a misdemeanour was adopted, continued to rely heavily on law enforcement. However, as each case study demonstrates, there is movement to provide evidence-based public health model services. In Asia, Africa and Europe the HIV epidemic drove a need to scale up treatment for injection drug use as a method of preventing spread of HIV and improving treatment adherence on HIV medications [69]. In South America, as in North America, the shift is driven by the negative impacts and poor outcomes of the war on drugs [70]. With differing precipitators and differing strategies, these countries are making efforts to change their approach.

All of the countries highlighted are low- or middle-income countries. Resources to address drug use are often primarily provided by donor countries, though the goal of all of the donor-driven activity is to develop sustainable infrastructure within each country.

## Conclusion

The international effort to address substance use in a humane and evidence-based manner is slowly finding its way into policy and practice. Even in countries where the approach has historically been focussed on militarized police action to reduce supply, or on inhumane treatment of people who use drugs, efforts to create an effective addiction public health infrastructure are in place. Aid organizations are providing direct care, particularly in response to the HIV epidemic, but also providing training and technical assistance to governments, organizations and individuals. Gradually a work force that will implement evidence-based prevention and treatment interventions is being built. While there is concern that the global COVID-19 pandemic may have provided cover for governments that want to restrict human rights [71], it has also provided opportunities to increase use of distance learning and provide treatment services *via* electronic communication. The transition may be permanent to some extent and provides an opportunity for broader reach and greater connection of educators and providers across country lines which may accelerate the dissemination of humane, evidence-based policy and practice to reduce the burden of drug use globally.

With the United Nations acceptance of a policy to change the way drug use is addressed, and resources provided by high-income countries and foundations a global movement has begun to improve care for people at risk of or with substance use disorders. The approach must be individualized to country and regional context, but the effort is broad and growing despite the barriers thrown up by pandemics, wars and other upheavals.

## Author contributions

Kimberly Johnson developed the concept, wrote the main sections and edited the full paper and agrees to be accountable for all aspects of the work. Irina Pinchuk wrote the Ukraine section and reviewed and edited the full paper, and approved the final submission. She agrees to be accountable for all aspects of the work. Marie Isabel Melgar wrote the Philippines section and reviewed and edited the full paper, and approved the final submission. She agrees to be accountable for all aspects of the work. Martin Osayande Agwogie wrote the Nigeria section and reviewed and edited the full paper and approved the final submission. He agrees to be accountable for all aspects of the work. Fernando Salazar Silva wrote the Peru section and reviewed and edited the full paper and approved the final submission He agrees to be accountable for all aspects of the work.

## Data Availability

Data sharing is not applicable to this article as no new data were created or analysed in this study.
